# Production of Recombinant Antimicrobial Polymeric Protein Beta Casein-E 50-52 and Its Antimicrobial Synergistic Effects Assessment with Thymol

**DOI:** 10.3390/molecules22060822

**Published:** 2017-05-31

**Authors:** Shohreh Fahimirad, Hamid Abtahi, Seyed Hadi Razavi, Houshang Alizadeh, Mansour Ghorbanpour

**Affiliations:** 1 Agriculture and Natural Resources Biotechnology Department, University of Tehran, Karaj 31587-11167, Iran; sh_fahimirad@yahoo.com (S.F.); srazavi@ut.ac.ir (S.H.R.); halizadeh@ut.ac.ir (H.A.); 2 Molecular and Medicine Research Center, Arak University of Medical Sciences, Arak, Molecular and Medicine Research Center, Arak University of Medical Sciences, Arak 38181-76941, Iran; 3 Department of Medicinal Plants, Faculty of Agriculture and Natural Resources, Arak University, Arak 3815688349, Iran; m-ghorbanpour@araku.ac.ir

**Keywords:** antimicrobial activity, *beta-*casein, E 50-52, synergism, thymol

## Abstract

Accelerating emergence of antimicrobial resistance among food pathogens and consumers’ increasing demands for preservative-free foods are two contemporary challenging aspects within the food industry. Antimicrobial packaging and the use of natural preservatives are promising solutions. In the present study, we used *beta*-casein—one of the primary self-assembly proteins in milk with a high polymeric film production capability—as a fusion partner for the recombinant expression of E 50-52 antimicrobial peptide in *Escherichia coli*. The *pET21a-BCN-E 50-52* construct was transformed to *E. coli BL21 (DE3)*, and protein expression was induced under optimized conditions. Purified protein obtained from nickel affinity chromatography was refolded under optimized dialysis circumstances and concentrated to 1600 µg/mL fusion protein by ultrafiltration. Antimicrobial activities of recombinant BCN-E 50-52 performed against *Escherichia coli*, *Salmonella typhimurium*, *Listeria monocytogenes*, *Staphylococcus aureus*, *Aspergillus flavus*, and *Candida albicans*. Subsequently, the synergistic effects of BCN-E 50-52 and thymol were assayed. Results of checkerboard tests showed strong synergistic activity between two compounds. Time–kill and growth kinetic studies indicated a sharp reduction of cell viability during the first period of exposure, and SEM (scanning electron microscope) results validated the severe destructive effects of BCN E 50-52 and thymol in combination on bacterial cells.

## 1. Introduction

The adverse impacts of chemical antimicrobial compounds as food preservatives on human health, growing food pathogen resistance to commonly-used antimicrobial agents, and increasing interest in natural food preservatives are the primary issues persuading researchers to find novel natural antimicrobial compounds as food preservatives [1]. Antimicrobial packaging systems serve as a strong obstacle for microbial agents, and extend food shelf life while diminishing preservative compound utilization. Therefore, novel antimicrobial polymers with natural sources have received considerable attention [2]. Bacteriocins are bacterial antimicrobial peptides which have been exploited as biopreservatives in the food industry for a hundred years [3]. Bacteriocins have been divided into four classes: Class II consists of three subgroups on the basis of their primary structure. The YGNGV-conserved motif, a disulphide bond linkage, and significant inhibitory impacts on *Listeria* sp. are most prominent in Class IIa bacteriocins, typically [4]. Bacteriocin E 50-52, with net charge of +2, produced by *Enterococcus faecium* belongs to class IIa bacteriocin [5]. The wide antibacterial spectrum, successful inhibition of antibiotic-resistant bacterial strains, heat stability, and the quality of being recognized as safe makes Bacteriocin E 50-52 a great candidate as a natural antimicrobial food preservative [6]. Bacteriocin derivation from natural bacterial sources is time-consuming, laborious, and costly. Solid-phase chemical synthesis provides small amounts of peptides, but biotechnological approaches are able to efficiently produce higher quantities [7]. However, using microbial hosts for the production of antimicrobial peptides leads to inconsiderable yield. 

The recombinant production of antimicrobial peptides in bacterial cells using different kinds of carriers has led to significant production [8]. *Beta*-casein (β-CN) is one of four main types of casein, a major milk protein [9]. β-CN’s homodimerization activity and amphiphilic structure cause the formation of micelles in aqueous solutions [10,11]. β-CN’s abilities in the formation of polymeric films were reported in separate studies [12,13,14].

In this study, we applied *beta*-casein as a carrier for the expression of E 50-52 in *E. coli* to create an antimicrobial polymeric monomer for further applications in food packaging. The antimicrobial activities of the fused BCN-E 50-52 against common food photogenes were assayed, and its synergistic effects with thymol as a food-grade monoterpene were calculated. 

## 2. Results

### 2.1. Expression of BCN E 50-52 in E. coli BL21

**Figure 1:**
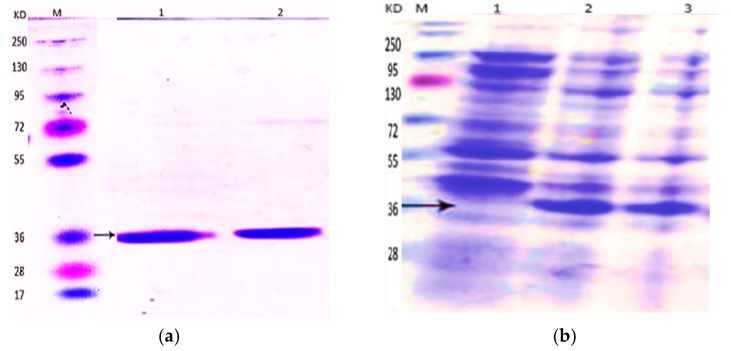
(**a**) Expression of BCN-E 50-52 in *E. coli BL21*. M: size marker; lane 1: total protein before induction; lane 2: total protein 2 h after induction; lane 3: total protein 24 h after induction. (**b**) Purification and refolding of BCN-E 50-52. M: size marker; lane 1: fusion protein retrieved by nickel affinity chromatography; lane 2: protein after refolding.

Protein production in various tested induction conditions was analyzed by SDS-PAGE. The greatest yield was achieved by applying 1.5× NB and 0.5 mM isopropyl thio β-d-galactosidase (IPTG). Overnight incubation of induced medium under 200 rpm shaking at 24 °C was the most convenient condition. The produced 35 kDa protein after 24 h and 2 h of induction are indicated by corresponding arrows in Figure 1a.

### 2.2. Purification and Refolding of BCN E 50-52

SDS-PAGE was used to analyze the quality and quantity of purified proteins by nickel nitrilotriacetic acid-agarose (Ni-NTA; Qiagen, Valencia, Spain; Alameda, CA, USA). Then, various changes in the durations and times of the purification steps were applied to gain much more purified protein without any extra bands (Figure 1b).

The best dialysis conditions were chosen based on the MIC (minimum inhibitory concentration) assay against *S. aureus* and *E. coli.* The results of dialysis in phosphate-buffered saline (PBS) buffers at different pHs revealed that low pH (pH = 4.5 and pH = 5) was the most effective for protein yields. Proteins was dialyzed in PBS-based exchanging buffers contained a complex of 0.5 mM cysteine and 0.05 mM cystine at pH = 4.5. BCN-E 50-52 concentration after the dialysis procedure was about 200 µg/mL, which was not sufficient for further assays. The problem was solved by utilizing Amicon centrifuging filters, yielding proteins with 1600 µg/mL concentration.

### 2.3. Antibacterial and Antifungal Activities

The MIC values of BCN-E 50-52 and thymol—alone and in combination—against the test bacteria and fungi are presented in Table 1.

**Figure 2:**
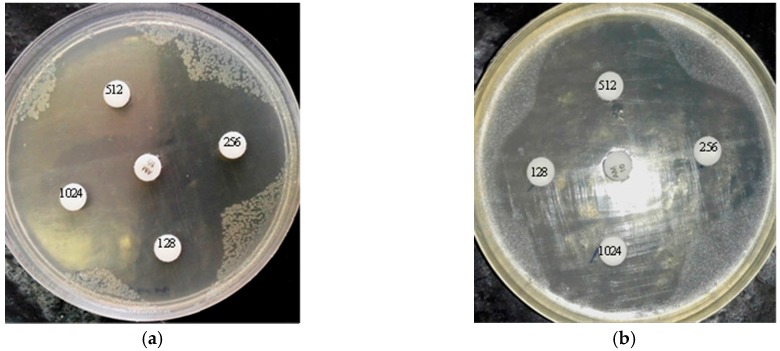

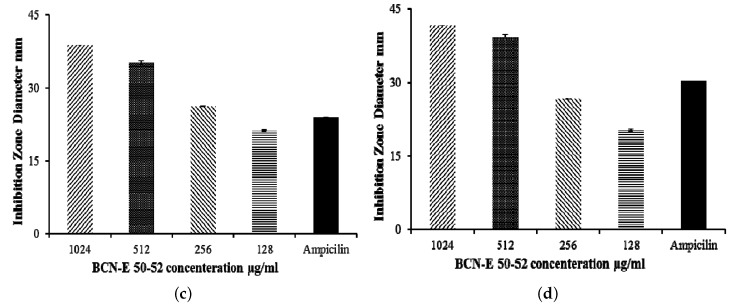
Agar disk diffusion of BCN E 50-52 against (**a**) *E. coli* and (**b**) *S. aureus.* BCN E 50-52 concentrations are written on disks in µg/mL. Inhibition zone diameters of BCN E 50-52 against (**c**) *E. coli* and (**d**) *S. aureus*.

The table demonstrates MIC values for each compound alone (subscript A for BCN E 50-52, and subscript B for thymol), the MIC value for each compound in combination (subscript C), the individual FIC (fractional inhibitory concentration) values, the combined FIC value (FIC_C_), and the synergism interpretation. The different test microorganisms illustrated distinct susceptibility to each compound individually and in combined forms. Overall, MIC tests indicated that BCN E 50-52 showed moderate antimicrobial activity against most tested microorganisms and no antimicrobial activity against *A. flavus*. Thymol (MIC 128 μg/mL) was the same for all tested microorganisms, except the MIC of 64 μg/mL for *A. flavus*. The combination of thymol and BCN E 50-52 devalued the BCN E 50-52 MIC against most of the tested organisms. The most intense synergisms between BCN E 50-52 and thymol were shown against *S. aureus* and *L. monocytogenes*, which recorded up to a 128-fold decline in the BCN E 50-52 MIC (the FIC_A_ value = 0.0078) (Figure 2). The FIC index reveals a kind of synergy between BCN E 50-52 and thymol in all test organisms, and no effects for *A. flavus* (Table 1).

### 2.4. Bactericidal and Fungicidal Activities

The results of the minimum bactericidal concentration (MBC) studies are summarized in Table 2, Table 3 and Table 4. The separate MBC tests of BCN E 50-52 and thymol against the tested bacteria indicated no bactericidal activity of BCN E 50-52 and a faint bactericidal activity of thymol. The calculated MBC (minimum bactericidal concentrations)/MIC values illustrated the bacteriostatic activity of both compounds against all test bacterial species. The resultant MFC (minimum fungicidal concentration)/MIC confirmed the fungistatic activity of each individual compound against *C. albicans* (Table 2).

The individual MBC values of each compound declined in combination tests. More than four-fold devaluation of BCN E 50-52 MBC was recorded against *E. coli*, *S. aureus*, and *L. monocytogenes* in combination with thymol than in individual assays (fractional bactericidal concentration, FBC = 0.25). MBC values of thymol were reduced two- to four-fold compared to the compound’s individual employment. BCN E 50-52 MFC values for *C. albicans* were reduced by more than two-fold, while there was no synergistic effect between thymol and BCN E 50-52 against *A. flavus* (Table 3). The synergistic effects of BCN E 50-52 and thymol are shown in Table 4. The FBCI (fractional bactericidal concentration index) and the interpretations for the activities of this combination against the test bacteria are recorded as synergistic and partial synergy.

### 2.5. Agar Disk Diffusion

Confirming the MIC results, the antimicrobial activity of BCN E 50-52 with different concentrations was assayed against *E. coli* and *S. aureus* (Figure 2). The test concentrations were equal to ½ MIC, MIC, 2× MIC, and 4× MIC for each bacterium. Diameters of the inhibition zones corresponding to different concentrations of BCN E 50-52 are shown in Figure 2. The qualitative data of agar disk diffusion after statistical analysis were compared to the control inhibition zone (ampicillin disk, 10 µg/mL) according to the CLSI recommendation standard. The disks containing 4× MIC, MIC, and 2× MIC concentrations of BCN E 50-52 against both bacteria created inhibition zones in the range of susceptibility. Both bacterial inhibition zones for ½ MIC concentration of BCN E 50-52 were assigned to the resistance range (Figure 2).

### 2.6. Time–Kill Curves

**Figure 3:**
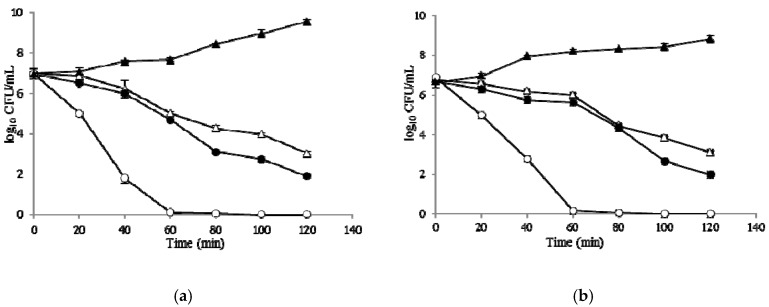
Bacterial-killing kinetics of BCN E 50-52 and thymol at 2× MIC against *E. coli* and *S. aureus.* (**a**) *E. coli*; control (filled triangles), BCN E 50-52 512 μg/mL (filled circles), thymol 256 μg/mL (open triangles), BCN E 50-52 512 μg/mL and thymol 256 μg/mL (open circles); (**b**) *S. aureus*; control (filled triangles), BCN E 50-52 512 μg/mL (filled circles), thymol 256 μg/mL (open triangles), BCN E 50-52 512 μg/mL and thymol 256 μg/mL (open circles).

The results of the time–kill assay for*E. coli* and *S. aureus* are presented in terms of the changes in the log_10_ CFU/mL of viable cells. Results of time–kill synergy studies are shown in Figure 3. The number of viable *E. coli* cells decreased sharply, with more than five reductions in log_10_ CFU mL^−1^ by the combination of BCN E 50-52 and thymol at 2× MIC within 40 min, and complete cell death occurred within 60 min. The test compound combinations achieved complete annihilation for *S. aureus* within 60 min, while BCN E 50-52 and thymol caused three and four reductions in log_10_ CFU mL^-1^ viable cells gradually for both test bacteria and could not inhibit the cell growth thoroughly within the first 120 min (Figure 3).

### 2.7. Growth Kinetic Curves

**Figure 4:**
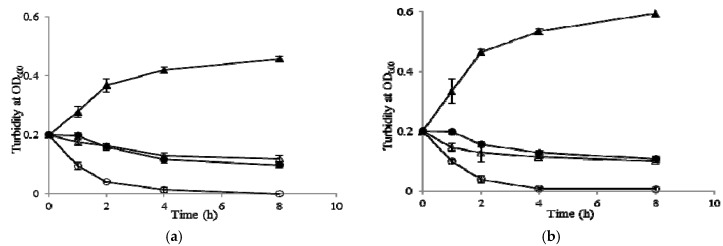
Bacterial growth kinetics of BCN E 50-52 and thymol at 2× MIC against *E. coli* and *S. aureus*. (**a**) *E. coli;* control (filled triangles), BCN E 50-52 512 μg/mL (filled circles), thymol 256 μg/mL (open triangles), BCN E 50-52 512 μg/mL and thymol 256 μg/mL (open circles); (**b**) *S. aureus*; control (filled triangles), BCN E 50-52 512 μg/mL (filled circles), thymol 256 μg/mL (open triangles), BCN E 50-52 512 μg/mL and thymol 256 μg/mL (open circles). OD: optical density.

In order to determine the mechanism of action of BCN E 50-52 against *S. aureus* and *E. coli*, the turbidity of bacterial cultures exposed to 2× MIC of BCN E 50-52 and thymol were recorded over time by a spectrophotometer. BCN E 50-52 and thymol separately caused more than 50% reductions in the turbidity of both bacterial suspensions after 4 h and maintained this rate for 8 h (Figure 4). The combination of BCN E 50-52 and thymol had much greater success in the reduction of more than 50% of *S. aureus* suspensions’ turbidity within the first hour, over 80% and 95%, declining after 2 h and 4 h, respectively. The *E. coli* suspension exposed to the combination of test compounds indicated 50%, 85%, and 95% density reductions occurring at 1, 2, and 4 h, respectively (Figure 4).

### 2.8. SEM Microscopy

*E. coli* cells treated with a combination of thymol and BCN E 50-52 at FIC values illustrated complete cell deformation and cell wall destruction after 2 h (Figure 4b). Indicating similar symptoms, membrane piercing and subsequent cytoplasmic material leakage were obvious morphological changes of *S. aureus* cells after treating for 2 h with thymol and BCN E 50-52 at FIC values (Figure 4d). These results confirm rapid antimicrobial activities of BCN E 50-52 in combination with thymol and validate the time–kill and growth curve results.

## 3. Discussion

**Figure 5:**
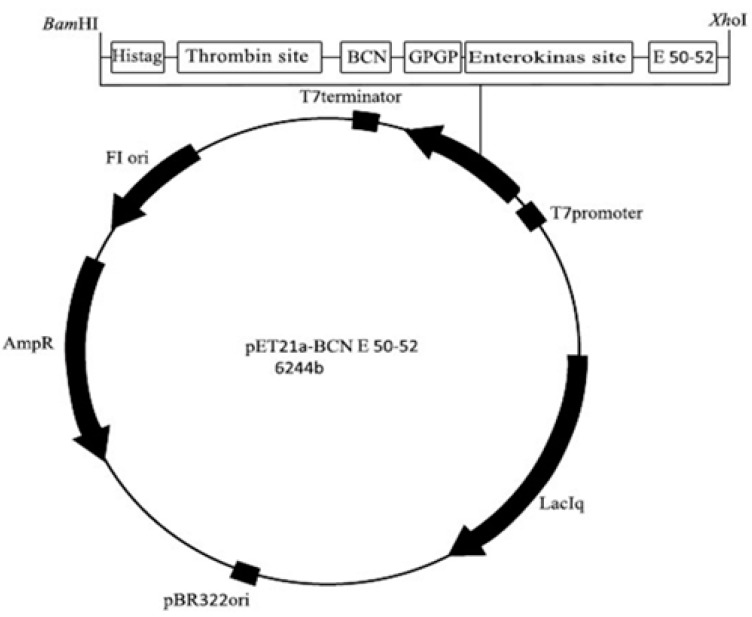
Design of the expression vector *pET21a-BCN E 50-52*. BCN: *beta* casein. GPGP: Double repeat of Glycine Proline as a flexible linker between two different proteins.

The emergence of a vast spectrum of resistance to common antimicrobial agents among microorganisms is turning into a unanimous global health concern. Research into new kinds of antimicrobial compounds is receiving much more attention. E 50-52, as a bacteriocin, is a food-grade strong antimicrobial peptide with diverse antimicrobial killing activities [5]. In addition to the development of antimicrobial edible films are active research fields in the food industry for employing natural sources as food preservatives [15]. *Beta*-casein’s ability to form edible polymeric films and attach to other polymers has been previously proved [16,17]. In the present study, we describe the use of *beta*-casein as a fusion partner for the expression of E 50-52 in *Escherichia coli BL21* (*DE3*) (Figure 5). It has been proved that the free N-terminal hydrophilic domain of Bacteriocin Class IIa plays the main role in attaching to bacterial cells and cell lysis process initiation [5,18]. β-CN fusing to the E 50-52 N-terminal decreased its antimicrobial activities and caused a more soluble expression of E 50-52 in a susceptible bacterial host. However, decreasing the IPTG ratio to 0.5 mM, reducing the incubation temperature to 24 °C, and extending the induction time to 24 h were the most efficient conditions for recombinant protein production (Figure 1). While there were significant decrements in E 50-52 antimicrobial intensity in the fusion to BCN, antimicrobial test results illustrated that BCN did not thoroughly hamper E 50-52 antimicrobial potency. Setting up the refolding process in an acidic buffer (pH = 4.5, 5) and applying the buffer including cysteine and cystin, followed by protein purification, boosted BCN E 50-52’s antimicrobial activation. In silico estimation of BCN E 50-52 charge over the pH range confirmed that the charge of BCN E 50-52 at low pH (4, 4.5, and 5) is much more positive (23.4, 15.9, and 9.1). The more positive net charge of recombinant BCN E 50-52 prompted its ability to attach to bacterial cell walls with negative charge [19]. There was accommodation between the disk diffusion-registered data and MIC results (Figure 2). Thus, bacteria were resistant to BCN E 50-52 sub-MIC, and the diameter of the inhibition zones increased according to ascending BCN E 50-52 concentration of the disks. Thymol is an important GRAS (generally recognized as safe) natural antimicrobial compound [20,21]. In order to promote BCN E 50-52 antimicrobial activities, the synergistic effects of this protein with thymol were assessed. The combination of the two compounds significantly devalued the compounds’ MIC and MBC (Table 1). The FICI and FBCI for most test microorganisms indicated at least additive efficacy (Table 1 and Table 4). Bacterial killing kinetics of BCN E 50-52, thymol, and their combination were conducted to survey the compounds’ pace of action during the first minutes of application. The accelerating force of thymol to BCN E 50-52 function was obvious (Figure 3).

**Figure 6:**
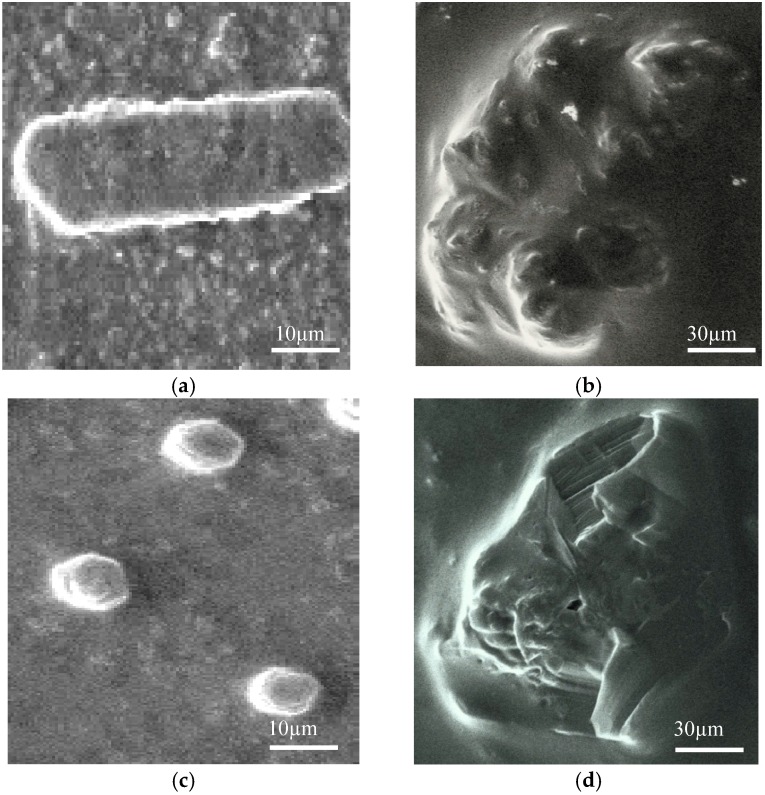
(**a**) SEM micrographs of untreated *E. coli*; (**b**) *E. coli* cell treated with a combination of BCN E 50-52 and thymol at FIC values; (**c**) SEM micrographs of untreated *S. aureus*; (**d**) *S. aureus* cell treated with a combination of BCN E 50-52 and thymol at FIC values.

Overall, all performed antimicrobial tests demonstrated substantial antimicrobial activities of BCN E 50-52 and thymol in combination with each other. Bacteriocins destabilize bacterial cells by piercing their membranes and, consequently cause cell lysis [5]. Thymol is capable of penetrating into the lipid assemblies and damaging lipid membranes [22]. Creating primary disorders in the cell membrane by each compound enhances their efficiency in expanding cell leakage with much lower concentrations. SEM analysis indicated drastic disruptions in the cell walls by piercing and rupturing of the outer membranes and severe lysis of cells incubated with BCN E 50-52 and thymol combinations at FIC values (Figure 6).

## 4. Materials and Methods

### 4.1. Materials and Chemicals

Ampicillin, *beta*-casein, brain-heart infusion media (BHIB and BHIA), dimethyl sulfoxide, Mueller Hinton media (MHB and MHA), RPMI-1640, Sabouraud dextrose agar, resazurin sodium salt, and thymol were purchased from Sigma Aldrich Co. (St. Louis, MO, USA). All other chemicals used were of analytical grade.

### 4.2. Bacterial Strains

The antibacterial activities were assessed against four different food spoilage microorganisms: Two Gram-negative strains, including *Escherichia coli ATCC 25922* and *Salmonella enterica serovar Typhimurium ATCC 14028*, and two Gram-positive strains, including *Listeria monocytogenes ATCC 19115* and *Staphylococcus aureus 29213*. Antifungal activity was evaluated against *Aspergillus flavus ATCC 204304* and *Candida albicans ATCC 76615*. *Escherichia coli BL21 (DE3)* was used as the recombinant protein expression host.

### 4.3. Construction of the Expression Vector pET21a-BCN E 50-52

The entire gene sequence, which contains the 5’ *Bam*H I, histidin tag, thrombin cleavage site, bovine *beta*-casein sequence (β-CN) (EMBL: M16645), GP linker, an enterokinase cleavage site, an E 50-52 corresponding sequence (UniProt: P85148), and *Xho* I 3’ were designed and codon-optimized for *E. coli* expression. The whole construct was synthesized by Biomatik Company (Cambridge, ON, Canada). The synthesized DNA (EMBL: LT795121) was inserted into the plasmid pET21a (Novagene) as the expression vector (Figure 6).

### 4.4. Expression of BCN E 50-52 in E. coli BL21

Competent *Escherichia coli BL21 (DE3)* were prepared based on standard protocols, and were transformed by *pET21a-BCN E 50-52* [23,24]. One of the recombinant colonies grown on plates containing ampicillin was incubated in 2 mL NB medium containing 100 μg/mL ampicillin at 37 °C. Then, 300 µL of overnight culture was inserted in 50 mL NB broth (100 μg/mL ampicillin), and incubated at 37 °C at 220 rpm. The cells’ optical density at 600 nm were measured occasionally, and when the culture turbidity was equal to 0.6, isopropyl thio β-d-galactosidase (IPTG) (1 mM) was added to induce protein expression. Four hours after induction, whole induced transformed bacteria were harvested by centrifugation at 5000 rpm for 20 min and the pellets were stored at −20 °C [25]. Various cultured media, different amounts of IPTG, alternative incubating temperatures, and different incubation times were tested to achieve the highest protein expression efficiency.

### 4.5. Purification of BCN E 50-52

Denaturing conditions using 8 M urea, followed by Ni-NTA agarose resin affinity chromatography (Qiagen, Alameda, CA, USA) were used for purification of the recombinant protein. SDS polyacrylamide gel electrophoresis (SDS-PAGE 12%) was applied to assay the quality of the purified recombinant BCN E 50-52 protein. The quantity of purified recombinant BCN E 50-52 protein was analyzed by the absorbance of 280 and 260 nm [26].

### 4.6. Refolding Optimization of BCN E 50-52

Using urea for protein purification denatures the active folding of proteins. So, the refolding process was done by applying prepared dialysis tubing, 10K molecular weight cut-off (MWCO) (Thermo Scientific SnakeSkin). Various pH (4.5, 5, 7, 8, 8.5) and different PBS-based exchanging buffers containing distinct amino acids (argenine, proline, and cysteine) were tested to gain the most efficient dialysis conditions [27]. The dialysis process was carried out at 4 °C for 24 h. The best dialysis conditions were chosen based on their MIC results.

### 4.7. Concentration of BCN E 50-52

A 10 kDa pore-size Amicon centrifugal filter (Merck Millipore, Darmstadt, Germany) was used to concentrate the dialyzed protein. 

### 4.8. Preparation of Standardized Inoculum

Bacterial inoculum preparation was done based on the CLSI standard protocol MO_7_-A_10_. Briefly, The selected isolated colonies were cultured in BHI broth and incubated at 37 °C until the culture optical density OD at 600 nm was 0.1 (1 × 10^8^ cells/mL). Then, the suspensions were diluted 1:100 with BHI to achieve 1 × 10^6^ CFU/mL [28].

### 4.9. Preparation of the Thymol Solution

Dimethyl sulfoxide (DMSO) was used as the thymol solvent at the final concentration of 1% (*v*/*v*). The initial concentration of thymol in solution was 1024 µg/mL [29].

### 4.10. Determination of Antibacterial Minimum Inhibitory Concentration (MIC)

CLSI protocol MO_7_-A_10_ was applied to calculate the MIC of BCN-E 50-52 and thymol against each tested organism [30]. Briefly, wells of the 96-well microplates from column 1:10 were filled by 50 μL of BHI broth. Then, 50 μL of the dialyzed recombinant 1024 µg/mL BCN-E 50-52 protein or 50 µL of thymol solution were diluted in wells by two-fold serial dilutions in separated 96-well plates. Subsequently, 50 μL of prepared bacterial inoculum was added to each well. Column 11, containing 100 μL of bacterial inoculum, was considered as the positive growth control, and Column 12, with 100 µL of the BHI, was the sterility control. After 24 h incubation at 37 °C, 20 μL of resazurin dyes (0.02% (*w*/*v*)) were added to each well and incubated again for 2 h [31]. The BCN-E 50-52 concentration in the last blue color well was scored as the MIC value. Ampicillin was used as the positive control, while 1024 μg/mL *beta*-casein solution was the negative control for BCN E 50-52 MIC assessment. Ampicillin and 1% DMSO were positive and negative controls for thymol MIC study, respectively. All experiments were performed in triplicate.

### 4.11. Determination of Minimum Bactericidal Concentration (MBC)

After MIC assessment, 100 µL of microplate blue wells corresponding to the MIC, and the above MIC values of BCN E 50-52 and thymol were plated on BHI agar. Following this, incubation of plates at 37 °C for 24 h was conducted to indicate the lowest BCN E 50-52 and thymol concentrations that led to no colony growth, which was considered as the MBC value. The antibacterial activities were defined based on the MBC/MIC ratio (MBC/MIC = 1 or 2 bactericidal, MBC/MIC = 4 or 16 bacteriostatic) [31].

### 4.12. Determination of Antifungal MIC

CLSI M_27_-A_4_ and M_38_-A_3_ protocols were used for antifungal susceptibility tests of BCN E 50-52 and thymol against *C. albicans* and *A. flavus* [32,33]. Briefly, 50 μL culture preparations in RPMI 1640 with 2% glucose were inoculated into the flat-bottom wells of 96-well microtiter plates. BCN E 50-52 and thymol, with initial concentrations 1024 μg/mL, were added to wells by a two-fold serial dilution. Then, 50 μL of 10^3^ CFU/mL prepared inoculum of *C. albicans* and 10^4^ CFU/mL conidiospores of *A. flavus* were poured per well. Plates were incubated at 35 °C for 48 h. Then, 20 µL of the resazurin solution was added to each well and the plate was re-incubated for 20 min. A change of color from blue (oxidized) to pink (reduced) indicated the growth of fungi [34,35]. The MIC was defined as the lowest concentration of each compound that prevented this change in color.

### 4.13. Determination of Minimum Fungicidal Concentration (MFC)

One-hundred microliters of the blue wells’ aliquots corresponding to the MIC and upper MIC values of BCN-E 50-52 and thymol were cultured on Sabouraud dextrose agar plates and incubated at 35 °C for 48 h. The lowest concentration that prevented visible growth was regarded as the MFC. The MFC/MIC ratio results were interpreted to fungistatic (MFC/MIC ≥ 4) or fungicidal activity (MFC/MIC < 4) of compounds [36].

### 4.14. Agar Disk Diffusion

The agar disc diffusion method was performed based on CLSI to assay the inhibition zone diameters of BCN-E 50-52 against *E. coli*, a Gram-negative bacteria, and *S. aureus*, a Gram-positive bacteria [37]. Briefly, blank disks (6 mm) were placed on prepared BHI agar plates inoculated with adjusted 0.5 McFarland turbidity inocula. Then, 20 µL of dialyzed BCN-E5 0-52 with different concentrations were poured on blank disks. Ampicillin (10 µg/mL) disks were placed on the center of plates for comparison. Inhibitory zones were measured after 24 h of incubation at 37 °C. All tests were performed in triplicate, and the mean of the inhibition diameters expressed were reported [38].

### 4.15. Synergy Study

A checkerboard dilution test was applied to evaluate the synergistic effects of BCN-E 50-52 and thymol against all tested microorganisms [20]. The fractional inhibitory concentration (FIC) was calculated according to the following formula: FIC of each drug (FIC) = (MIC of drug in combination)/(its MIC value). The FIC index (FICI) values were calculated using the following equation: FICI = FIC_A_ + FIC_B_ [20].

The interpretation is as follows: synergy, <0.5; partial synergy, 0.5–0.75; additive effect, 0.76–1.0; indifference, >1.0–4.0; and antagonism, >4.0 [39,40]. The fractional bactericidal concentration index (FBCI) is the sum of the FBCs of each of the compounds and is calculated and interpreted the same as mentioned above for FIC [41].

### 4.16. Time–Kill Curves

A time–kill assay was performed on 10 mL of *E. coli* as an important Gram-negative bacteria, and *S. aureus*, as the most common Gram-positive bacteria in food poisoning, with a starting inoculum of 10^6^ CFU/mL in the exponential phase. BCN-E 50-52 and thymol with 2× MIC concentrations alone or in combination were applied for investigation of their single and combination impacts on cell viability. Samples were taken at 0, 20, 40, 60, 80, 100, and 120 min after incubation and plated on BHI agar for colony counting. The detraction pattern of viable bacteria cell numbers and synergistic effects were determined after 24 h of incubation at 37 °C [42]. These experiments were performed in duplicate. Positive controls for the assay included ampicillin (10 µg/mL), and bacterial cultures without any agents were included as negative controls [43,44].

A reduction of ≥1 log_10_ relative to the initial inoculum indicates antimicrobial activity. In the synergism assay, a reduction of ≥2 log_10_ and 1 ≤ log_10_ ≤ 2 were considered as synergistic and additive, respectively [41].

### 4.17. Growth Curve

In short, mid-log phase bacterial cultures with a turbidity of 0.6 at 600 nm were diluted in MHB, achieving an OD600 of = 0.2 (10^8^ CFU/mL). After that, 200 µL of bacterial cultures were allocated to separate culture tubes and 2× MIC concentrations of BCN-E 50-52 and thymol alone or in combination were added to particular tubes. Tubes were incubated at 37 °C. At specific time intervals, turbidity was measured at 600 nm. The assay was carried out in duplicate [40].

### 4.18. SEM Microscopy

Overnight cultures of *E. coli* and *S. aureus* were adjusted to OD 0.1 (1 × 10^8^ cells/mL) at 600 nm and were then diluted with BHI medium to achieve 1 × 10^6^ CFU/mL cell density. Afterwards, bacterial cells were treated with thymol and BCN-E 50-52 at the determined FIC values for 2 h at 37 °C. Untreated controls were prepared in BHI medium with the same cell density. The bacterial cells were centrifuged at 12,000× *g* for 15 min, then washed two or three times and resuspended in sterile PBS. Ten microliters of suspension was spread onto a microscope slide and fixed in 2.5% glutaraldehyde. Then, samples were coated with gold. The changes in cell morphology were analyzed by SEM (AIS2100, Seron Technologies, Uiwang-si, Gyeonggi-do, Korea) [45,46].

### 4.19. Statistical Analysis

All statistical analyses were performed using SPSS (Version 16.0; SPSS, Inc., Chicago, IL, USA) software. Data analysis was presented as means ± standard deviations. Statistical significance was defined as *p* < 0.05. Mean values analysis was calculated by a Tukey test at the 0.05 level of significance. All related graphs were created with Microsoft Excel 2010.

## Figures and Tables

**Table 1:** The minimum inhibitory concentrations (MICs) of BCN-E 50-52, thymol, and their combinations against different microorganisms.

Microorganism	MIC (μg/mL)							
	(BCN-E 50-52)_A_	(BCN-E 50-52)_C_	FIC_A_	Thymol_B_	Thymol_c_	FIC_B_	FIC_C_	Interpretation
**Gram-negative**								
*E. coli*	25	8	0.031	128	16	0.125	0.156	synergistic
*S. typhimurium*	256	4	0.015	128	32	0.25	0.265	synergistic
**Gram-positive**								
*S. aureus*	256	2	0.0078	128	16	0.125	0.132	synergistic
*L. monocytogenes*	256	2	0.0078	128	64	0.5	0.507	partial synergy
**Fungi**								
*C. albicans*	256	8	0.031	64	32	0.5	0.515	partial synergy
*A. flavus*	-	-	-	128	64	0.5	-	non-synergistic

Values represent mean of three replications. FIC: fractional inhibitory concentration; Subscript A indicates BCN E 50-52; Subscript B indicates thymol; Subscript C indicates each compound in combination.

**Table 2:** The minimum bactericidal concentrations and the minimum fungicidal concentrations of BCN E 50-52 and thymol.

Microorganism	Separated Studies					
	(BCN E 50-52)_A_		Interpretation	Thymol_B_		Interpretation
	MBC	MBC/MIC		MBC	MBC/MIC	
**Gram-negative**						
*E. coli*	>512	4	bacteriostatic	512	4	bacteriostatic
*S. typhimurium*	>512	>4	bacteriostatic	>512	>4	bacteriostatic
**Gram-positive**						
*S. aureus*	>512	8	bacteriostatic	512	4	bacteriostatic
*L. monocytogenes*	>512	>4	bacteriostatic	512	4	bacteriostatic
**Fungi**						
	**MFC**	**MFC/MIC**		**MFC**	**MFC/MIC**	
*C. albicans*	>512	>4	fungistatic	256	4	fungistatic
*A. flavus*	-	-	No effects	256	4	fungistatic

Values represent the mean of three replications. MBC: minimum bactericidal concentration; MFC: minimum fungicidal concentration.

**Table 3:** The minimum bactericidal concentrations and the minimum fungicidal concentrations of BCN E 50-52 and thymol in combination.

Microorganism	Synergism Study					
	(BCN E 50-52)_c_		Interpretation	Thymol_c_		Interpretation
	MBC	MBC/MIC		MBC	MBC/MIC	
**Gram-negative**						
*E. coli*	128	16	bacteriostatic	128	8	bactericidal
*S. typhimurium*	128	64	bacteriostatic	128	4	bacteriostatic
**Gram-positive**						
*S. aureus*	16	64	bacteriostatic	128	4	bacteriostatic
*L. monocytogenes*	128	64	bacteriostatic	256	8	bacteriostatic
**Fungi**						
	****	**MFC/MIC**		**MFC/MIC**	****	
*C. albicans*	256	32	fungistatic	256	16	fungistatic
*A. flavus*	-	-	-	256	4	fungistatic

Values represent the mean of three replications. MBC: minimum bactericidal concentration; MFC: minimum fungicidal concentration.

**Table 4:** The fractional bactericidal concentration index (FBCI) of synergism study.

Organism	FBC			Interpretation
	BCN E 50-52_c_	Thymol_C_	FBCI	
**Gram-negative**				
*E. coli*	<0.25	0.25	<0.5	partial synergy
*S. typhimurium*	<0.25	< 0.25	<0.5	partial synergy
**Gram-positive**				
*S. aureus*	<0.25	0.25	<0.5	partial synergy
*L. monocytogenes*	<0.25	0.5	<0.75	partial synergy
**Fungi**				
*C. albicans*	<0.5	1	<1.5	no synergy
*A. flavus*	-	1	-	no synergy

Values represent the mean of three replications.
